# Supplementary material for: THiCweed: fast, sensitive detection of sequence features by clustering big datasets
Source: Nucleic Acids Res. 2017 Dec 18;46(5):e29. doi: 10.1093/nar/gkx1251 (PMC5861420; doi:10.1093/nar/gkx1251)
Supplement: Supplementary Data [file gkx1251_supp.pdf]

THiCweed: fast, sensitive detection of sequence features by clustering big data sets
Supplementary information

Ankit Agrawal [1], Snehal V. Sambare [1], Leelavati Narlikar [2], and Rahul Siddharthan [1*]
[1] *The Institute of Mathematical Sciences (HBNI), Chennai 600113, India*
[2] *CSIR National Chemical Laboratory, Pune 411008, India*

October 16, 2017

Significance criteria

The likelihood of the windows in a cluster being all sampled from the same position weight matrices is

$$P(C) = \prod_{i=1}^{W} \frac{\prod_\alpha \Gamma(n_{i\alpha} + c)\Gamma(4c)}{\Gamma(\sum_\alpha n_{i\alpha} + 4c)\Gamma(c)^4} \quad (1)$$

where W is the length of the window, $\alpha =$ one of the nucleotides `A`, `C`, `G` or `T`, $n_{i\alpha}$ is the number of occurrences of nucleotide α at position i in the cluster, and c is a pseudocount (0.5 here). When a cluster C is split in two clusters C_1 and C_2, a measure of the quality of the split is the log-likelihood ratio $\log(P(C_1)P(C_2)/P(C))$ of the sequences being sampled from different PWMs versus their being sampled from the same PWM.

Merely asking for this quantity to be positive is insufficient. Suppose the sequences are random, but we split the cluster such that all sequences containing A or T at a particular position j end up in one clusters, sequences containing C or G in the other. This is always possible and gives a positive LLR, but is not significant. Call the LLR in this case L_1.

Suppose, instead, that this split is possible on a *pair* of positions j and k: after the split, nucleotides at these positions in one cluster are always A and T and in the other are C and G. This is not possible in general and, if possible, is certainly significant. Call the LLR in this case L_2.

Setting L_2 as the threshold for significance may be too stringent: instead we consider a threshold

$$L_T = L_1 + T(L_2 - L_1)$$

where T is an adjustable parameter, and demand that the LLR of the actual split exceed L_T.

A second significance criterion is demanding that splits are reproducible. We split C into C_1 and C_2, re-join them, and re-split into C_1' and C_2'; in general the results are different since the splits start from randomized clusters. The similarity of the splits is evaluated using the adjusted Rand index ("ARI") (Hubert and Arabie, 1985). We carry out this split four times and demand that three of the six resulting pairwise comparisons have an ARI greater than a threshold r ($r = 0$ indicating near-random splits, and $r = 1$ indicating perfect agreement). If the three pairwise comparisons of the first three splits already exceed this threshold, the fourth is not carried out.

The figure on the next page shows results on the synthetic data (1000bp set) of THiCweed runs with various choices of T and r. While some extreme choices are detrimental to performance (presumably because they encourage excessive insignificant splitting or discourage valid splitting), the performance overall is not extremely sensitive to the choices of T and r. Based on this figure, the choice of $T = 0.4$, $r = 0.2$ are set as defaults for the program and used in the benchmarks for the paper, but the user may make other choices via the command line.

Reference:
Hubert,L. and Arabie,P. (1985) Comparing partitions. *Journal of classification,* **2**(1), 193–218.

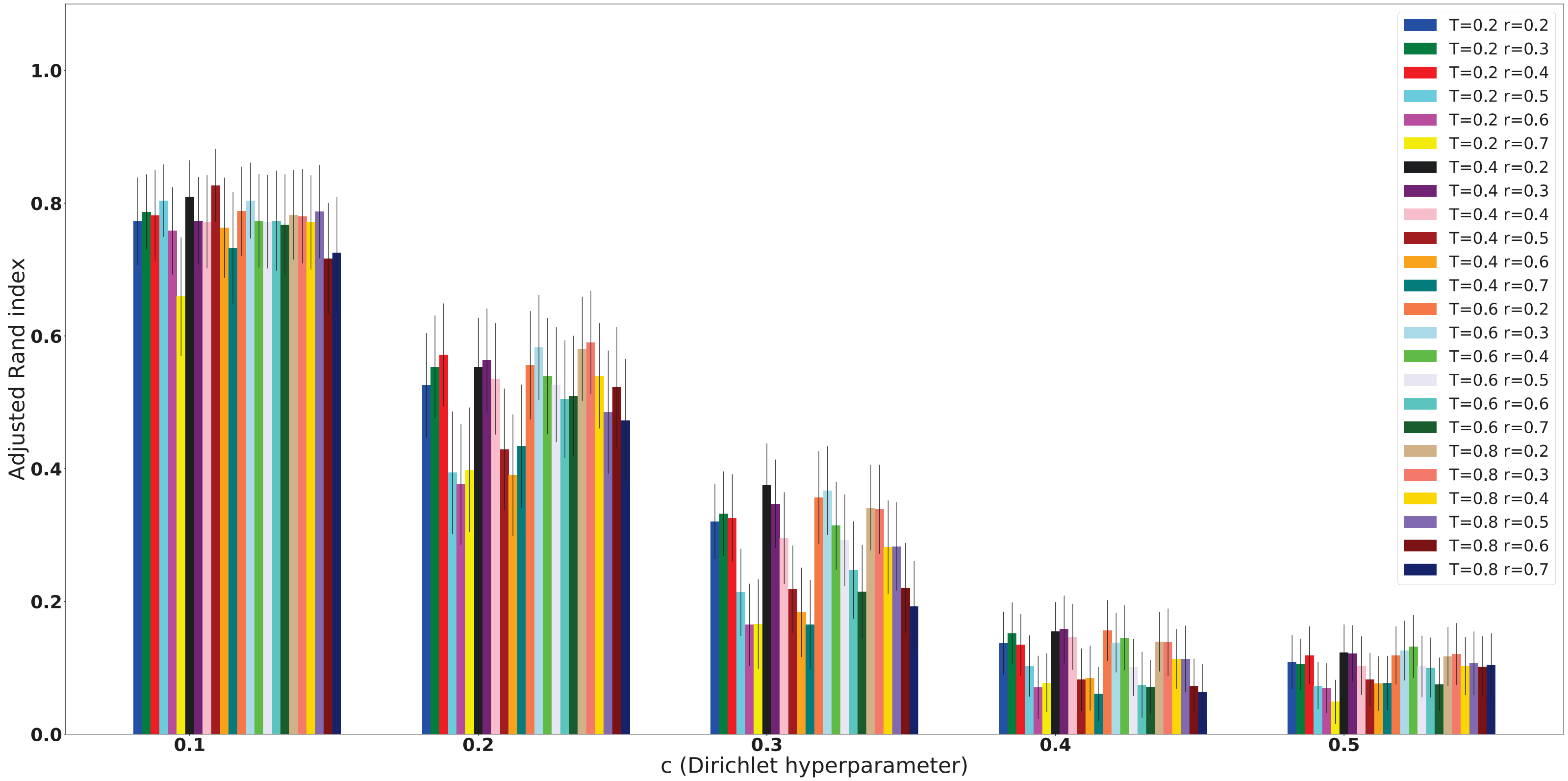

Performance of THiCweed on synthetic data, 1000bp. Compare with figure 2(b) of main manuscript.
